# Safety and Aesthetics of Autologous Dermis-Fat Graft after Parotidectomy: A Multidisciplinary Retrospective Study

**DOI:** 10.3390/jpm13081200

**Published:** 2023-07-28

**Authors:** Ciro Emiliano Boschetti, Rita Vitagliano, Nicola Cornacchini, Mario Santagata, Valentina Caliendo, Maria Paola Belfiore, Giuseppe Colella, Gianpaolo Tartaro, Salvatore Cappabianca

**Affiliations:** 1 Oral and Maxillofacial Surgery Unit, Multidisciplinary Department of Medical-Surgical and Dental Specialties, University of Campania “Luigi Vanvitelli”, 80131 Naples, Italy; ciroemiliano.boschetti@unicampania.it (C.E.B.); nicola.cornacchini@studenti.unicampania.it (N.C.); mario.santagata@unicampania.it (M.S.); giuseppe.colella@unicampania.it (G.C.); gianpaolo.tartaro@unicampania.it (G.T.); 2 Department of Precision Medicine, University of Campania Luigi Vanvitelli, Piazza Luigi Miraglia, 80138 Naples, Italy; valentina.caliendo@unicampania.it (V.C.); mariapaola.belfiore@unicampania.it (M.P.B.); salvatore.cappabianca@unicampania.it (S.C.)

**Keywords:** reconstructive surgery, oncologic head and neck surgery, fat graft, parotidectomy, head and neck MRI

## Abstract

(1) Background: In surgical procedures for maxillofacial tumours, it is challenging to preserve functional and cosmetic properties in the affected patients. The use of fat grafting is considered as a valuable alternative to overcome postoperative aesthetic asymmetry problems. (2) Methods: In this study, we enrolled thirty patients with parotid gland tumours in which a partial or complete parotidectomy was performed with positioning in the parotid bed of autologous dermis-fat grafts. We evaluated the satisfaction rate of the patients and the objective efficacy in solving the deformity by comparing MRI data before and after surgery. (3) Results: Twenty-six patients showed a satisfying cosmetic result with proper facial symmetry between the affected side and the healthy one. Two patients presented mild postsurgical complications such as haematomas, and two patients reported temporary weakness of the facial nerve related to the parotidectomy. (4) Conclusions: Based on the imaging data obtained via MRI before and after surgery, we can assess that the employment of fat grafts in parotidectomy surgical procedures gives good cosmetic results and does not affect the post operative management and follow up of oncologic patients.

## 1. Introduction

Parotid gland tumours are considered a medical condition that, most of the time, requires surgical treatment by performing a partial or complete parotidectomy.

This surgical procedure represents a challenge for head and neck surgeons because of the anatomical location of the parotid gland. Some tumours are literally surrounded by a complex network of facial nerves branches, and the main difficulty is the performance of a tumour resection while avoiding nervous lesions and preserving nervous integrity and functionality as much as possible. However, due to the anatomical, aesthetical, and functional complexity of the region, there are important cosmetic implications after the removal of a parotid gland tumour, as the resectioning of parotid tumours has been shown to cause facial depression deformities [1]. As a matter of fact, many parotid tumours could reach massive dimensions, causing not only a concern for the general behaviour of the tumour and its tempestive eradication but also causing a visible facial deformity and asymmetry that would negatively affect patients’ social lives [2]. When a parotid gland tumour with relevant dimensions is removed, it leaves a depression in the preauricular and infra-auricular region that could create facial deformities and asymmetry and could potentially create aesthetical discomfort in the patients after surgery [3]. In order to avoid a poor cosmetic result that may severely decrease a patient’s quality of life and self-esteem, especially in young patients [4], the evaluation of a quick, safe, satisfactory, and cost-effective reconstructive option could be necessary. Thus, according to a few authors, this reconstructive procedure should be performed during the same operating session immediately after the tumour resection, as implanting grafts in the affected anatomical area could immediately overcome any cosmetic defect; also, a single parotidectomy and a parotidectomy with the harvesting and implanting of a reconstructive graft have similar operation times [1,5,6]. Different types of materials and grafts can be employed for the reconstruction of facial soft tissue defects, such as alloplastic materials (polyethylene, silicone, or polyacrylamide), or grafts, such as superficial temporal fascia flaps, sternocleidomastoid muscle flaps [7], superficial muscular aponeurotic system (SMAS) flaps, and free fat grafts [8,9]. In our study, in all patients, the postresection defect was treated with free autologous dermis-fat grafts.

Our multidisciplinary study aimed to demonstrate, in our experience, how this century-old milestone in craniofacial reconstructive surgery has a complete oncological safety profile during the surgery and for the oncological follow up, and it is also a reliable procedure for surgeons, without the lengthening of surgical times, and has a low rate of complications and a high aesthetical satisfaction rate for the patients.

## 2. Materials and Methods

**Figure 1:**
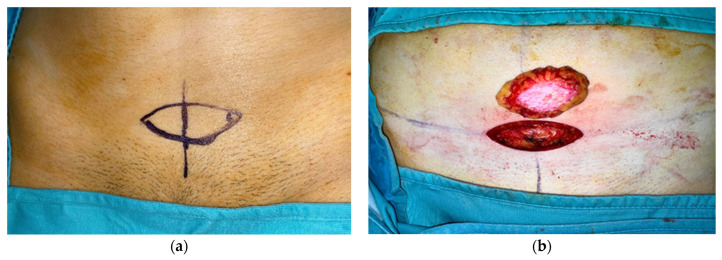
The en bloc fat graft design and harvesting. (**a**) Patient 1. Surgical design of the en bloc fat graft with a linear sovrapubic incision. (**b**) Patient 2. Surgical harvesting of the en bloc autologous fat graft after de-epithelialisation process.

**Figure 2:**
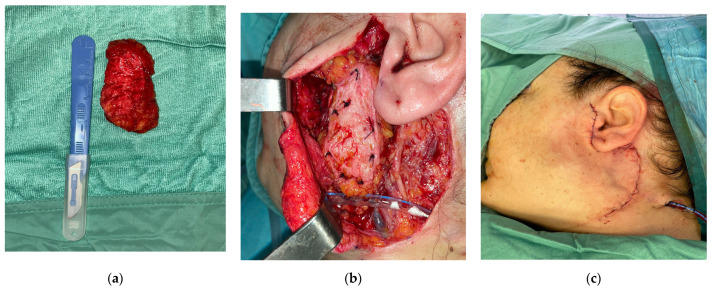
The en bloc fat graft design and harvesting. (**a**) Patient 1. En bloc fat graft obtained from the sovrapubic region after the de-epithelialisation process. (**b**) Patient 1. Surgical fixation process of the en bloc autologous fat graft to the surgical gap after parotidectomy. (**c**) Patient 1. Closure of the surgical site after the fixation process of the en bloc autologous fat graft and the placement of the drainage.

In our study, thirty patients with parotid gland tumours were enrolled from February 2019 to May 2022, who received a partial or complete parotidectomy with contemporary reconstruction with autologous en bloc dermal fat grafts. Twenty-two of these patients had a benign lesion, and the remaining eight patients had a malignant tumour. The surgical procedure consisted of a classic Blair incision with the sub-surface dissection of the musculoaponeurotic system, the identification of the tumour and its isolation from the surrounding nerve structures, tumour resection, and en bloc dermal fat grafting. The facial nerve trunk and its branches were found and preserved in each case. In all cases, we preferred to obtain the en bloc dermal fat graft through a linear suprapubic incision with a minimum length of 6 cm, which would be individually shaped according to the size of the defect of each patient, and we carefully closed the surgical access with cosmetic intradermal sutures with no drainage. After the harvesting process of the autologous en bloc dermal fat graft from the suprapubic region (Figure 1), the graft was irrigated with antibiotic-soaked saline and contoured with scissors to fit the anatomical region and cover the defect. A ‘monobloc’ de-epithelialisation technique was performed, involving the removal of the epidermal layer as a whole with a scalpel, on the en bloc autologous dermal fat graft in all cases (Figure 2a) [10].

After the shaping process of the autologous fat graft according to the size of the defect and its positioning in the postparotidectomy surgical gap, we fixated it to the parotid residual bed with absorbable sutures (Figure 2b); the SMAS flap was used as a cover of the dermis-fat graft to give a vascular supply, and then, we placed a drainage in the parotid site for a minimum of 24 h, considering less than 20 mL of serosanguineous fluid an indication for its removal (Figure 2c).

The postoperative management included local cooling application, 3 days of antibiotic therapy, and 20 days of therapy with dietary supplements composed of escin and bromelain supplements (Floganday^®^, Maya Pharma, Naples, Italy) in order to contain the oedema. Exclusion criteria were patients who had preoperative planning of neck dissection, in order to avoid the risk of an increase in perioperative and postoperative complications, and patients over 80 years of age. The whole procedure for obtaining the en bloc autologous dermis-fat graft was performed simultaneously to the parotidectomy and it did not represent a time-prolongating factor for the whole surgical session. MRI was performed before the surgery, and 60 days, 6 months, and one year after surgery for the oncological follow up, to determine proper facial symmetry and tissue density to achieve a satisfying cosmetic result. No touch-up procedures were performed after surgery. With data obtained with MRI, we were able to perform mirroring by superimposing images before and after surgery and objectively assessing the effective compensation of the defect with dermis-fat grafting, and in the long-term follow up, we approximately evaluated whether there was fat reabsorption.

## 3. Results

Thirty patients, twenty females and ten males, were enrolled in this study. Their age ranged from 14 to 78 years old. All patients were Caucasian, except one patient, who was of African descent. Twenty-two patients had benign lesions, and eight had malignant lesions (Table 1).

**Figure 3:**
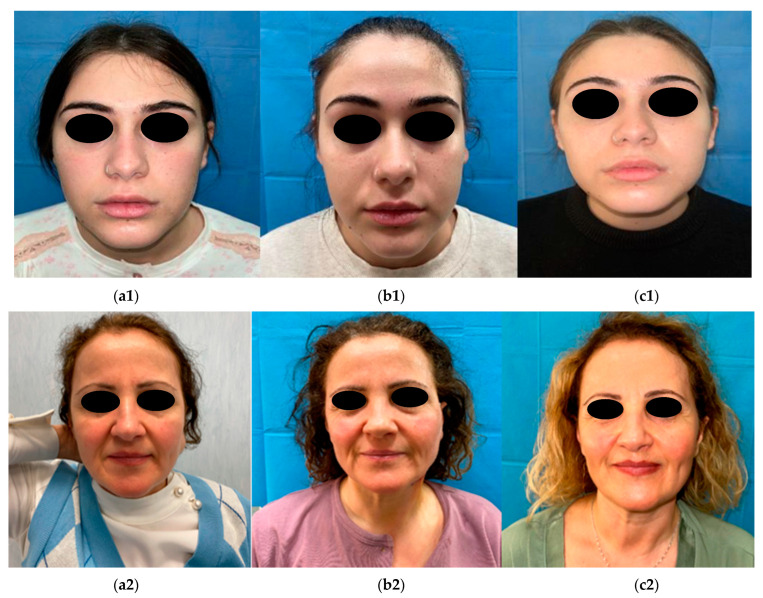
Sequence of clinical assessment of facial symmetry before surgery (**a1**,**a2**), 7 days after surgery (**b1**,**b2**), and during 3-month follow-up after surgery (**c1**,**c2**) in patient 1 (**upper** row) and patient 2 (**lower** row).

Tumours were resected without damaging the surrounding facial nerve branches, only in five cases were the ipsilateral facial nerve or its inferior branches (the marginalis mandibulae or the buccalis branches) strictly adherent to the neoplasms and correctly isolated without direct damage to the branches of the facial nerve. In all cases, the en bloc dermis-fat graft was obtained via a suprapubic incision; in three female patients, the incision was performed on a previously existing scar. The hospitalization time for all the patients was three days. The short-term follow up was performed after three days, seven days, ten days, and fourteen days after hospital discharge. The long-term follow up was performed after three, six, and twelve months. Two patients reported, as an early complication, haematoma in the parotidectomy area after the surgery in the first week after surgery; in both cases, this complication was successfully treated with light compressive treatment and escin and bromelain ointment (Floganday^®^) three times per day for 2 weeks. One patient reported facial nerve weakness related to the surgical procedure used for the removal of the tumour, which was strictly adherent to the network of several facial nerve branches; it involved the mandibular branch and completely resolved over a maximal duration of one month, with the application of Kabat therapy [11] and the use of oral corticosteroid therapy and vitamin “B-complex” supplements [12]. We did not observe complications with the fat graft such as infections, liquefaction, or graft loss due to excessive fat resorption [13,14]. We asked the patients to answer a satisfaction questionnaire with a score ranging from 0 to 5. According to the patients that decided to participate in our questionnaire, twelve patients gave a score of 5/5; four patients gave a score of 4/5; three patients gave a score of 3/5; two patients gave a score of 2/5; one patient gave a score of 1/5. We documented the preoperative and postoperative courses of patients through frontal facial, lateral facial, and semi-lateral facial pictures, and with these pictures, we reported the facial expression before and after surgery to assess nervous integrity (Figure 3).

## 4. Discussion

The German surgeon Gustav Neuber was the first to describe and use a free fat autografting technique in 1893 [15]. He transplanted adipose tissue from a patient’s arm to the orbit to cosmetically improve the sequelae associated with osteomyelitis. Since then, fat grafting has become a progressively employed technique for reconstructive surgery in maxillofacial procedures, like, for example, in case of facial contouring, radiation damage, burn injuries, parotidectomies, or the surgical treatment of MRONJ [16,17]. Fat tissue has a very similar texture and consistency to the parotid tissue [1], representing an ideal substitute in the case of a partial or complete parotidectomy. The donor sites are usually covered body parts such as the abdominal, gluteal, iliac, or sacral region. The final decision about the donor site for an en bloc autologous dermis-fat graft is influenced by the fat volume required, relying on the first-step surgical procedure applied (partial or complete parotidectomy); then, the fat volume of the donor site based on the location of excess adipose tissue is evaluated for each patient case-by-case and is based on both surgeon and patient preferences. In our patients, we always obtained the en bloc autologous dermis-fat graft with a linear incision from the suprapubic lower abdominal region because it is a body part usually easily covered by clothes or underwear, it has easy and fast surgical access, and it also leaves a minimally visible scar with a cosmetic intradermic suture. It is also a saving-time reconstructive option that does not increase the length of surgery. The time taken to harvest and de-epthelially engraft the en bloc autologous dermis fat graft, in our cases, was always about forty-five minutes, and it could be performed simultaneously to the parotidectomy; the autologous en bloc fat graft, as a low-complexity technique, could also easily be a suitable reconstructive alternative for patients with comorbidities and poor systemic conditions [18]. The en bloc autologous dermis-fat graft technique is also very useful to prevent a few postoperative complications of parotidectomy, like Frey’s Syndrome or facial haematomas or blood loss [19,20].

We also found that in three patients who underwent an autologous fat graft who had a mild facial nerve injury (HB scale I-II), with the help of our protocol of Kabat therapy [21], they had a faster recovery time for the weakness of the facial nerve, plausibly related to the protective effect of the fat graft on the nerve; according to few authors, this could be correlated to native adipose-derived stem cells present in the transferred tissue, which could potentially act upon regenerating axons, but conclusions are still not completely clear, and studies are still in progress (Figure 3) [22].

Considering the reabsorption rate of fat grafting, we collected more fat with an over-approximation between 20 and 30%. Complications associated with parotidectomy reconstruction with a fat graft are haematoma in the donor site, fat graft necrosis and liquefaction, surgical sutures’ dehiscence, cutaneous complications in the donor site such as itching, hypoesthesia, and hypersensitivity [17]. The most unpredictable complication associated with a fat graft is the rate of resorption in the postoperative period [14]. This resorption can potentially range from 0% to 100%, making the whole procedure have unsatisfactory results, because if the reabsorbed fat is excessive, the depression is not compensated for, and aesthetic symmetry not respected. In our study, patients with malignant lesions were involved, because, based on the recommendations of our radio diagnostic team, fat tissue is clearly distinguishable from malignant tumour tissue in MRI [3,5], dispelling the concept that fat grafting should be avoided in patients with malignancies because the reconstruction can mask the tumour identification in postoperative diagnostic imaging. This gave us the “green light” to proceed with fat grafting on patients with malignant tumours.

### 4.1. The Radiologist’s Perspective

#### 4.1.1. MRI Protocol

All patients underwent contrast-enhanced MRI of the face and neck before and after parotidectomy.

MRI examinations were performed on a 1.5 T scanner (SIGNA Voyager, GE Healthcare, Chicago, IL, USA) using a phased-array head and neck coil. The MRI protocol consisted of T1-weighted (T1W) fast spin echo (FSE) (TR/TE: 551.0/9.2 ms), T2-weighted (T2W) fast spin echo (FSE) (TR/TE: 8994.0/83.1 ms), and T2W STIR (TR/TE: 14,878.0/68.6 ms) sequences in the axial plane with 3 mm slice thickness.

DWI was performed with an echoplanar SE sequence (TR/TE, 14,782/66.8 ms; FOV, 22 × 22 cm^2^; matrix, 128 × 128; NEX, 2; slice thickness, 3 mm) in the axial plane with two different b-values (0 and 1000 s/mm^2^). Finally, fat-saturated isotropic 3D T1w GRE sequences (LAVA) on the axial plane were obtained before and after the intravenous injection of 0.2 mL/kg of Gadoteric Acid (Claricyclic, GE Healthcare, Chicago, IL, USA) at 2.0 mL/s. Multiplanar reconstructions on the coronal and sagittal plane were subsequently performed.

In postoperative studies, the volume of the reconstructed parotid gland was determined through semi-automatic segmentation at the workstation (aycan Worskstation, aycan Medical Systems, Rochester, New York, USA). Finally, a volumetric comparison with the contralateral parotid gland was performed.

#### 4.1.2. MRI Analysis

MR image analysis was performed by two radiologists with >2 years of experience in head and neck radiology.

**Figure 4:**
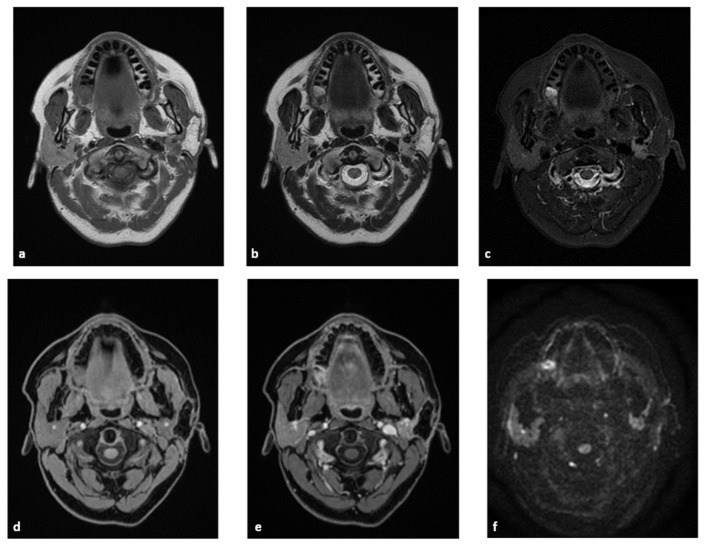
Female, 53 years old. First postoperative MR examination after partial parotidectomy with dermal fat graft. Fat appears hyperintense on both FSE T1-weighted (**a**) and T2-weighted (**b**) axial images and homogenously hypointense on STIR images (**c**). The fat graft shows no significant enhancement between pre- (**d**) and postcontrast (**e**) acquisitions and appears hypointense on DWI at b-1000 (**f**).

Fat has an unequivocal appearance on MRI, as it shows high signal intensity on spin echo T1- and T2-weighted images and rather homogeneous signal loss on fat-suppressed sequences, with no contrast enhancement or restricted diffusion. Thanks to these features, dermal fat grafts were easily identified in all the postoperative studies (Figure 4).

Differential diagnosis with tumour recurrence was also feasible, as the latter shows an intermediate T1-weighted signal and, most importantly, a variable degree of contrast enhancement and restricted diffusion.

**Figure 5:**
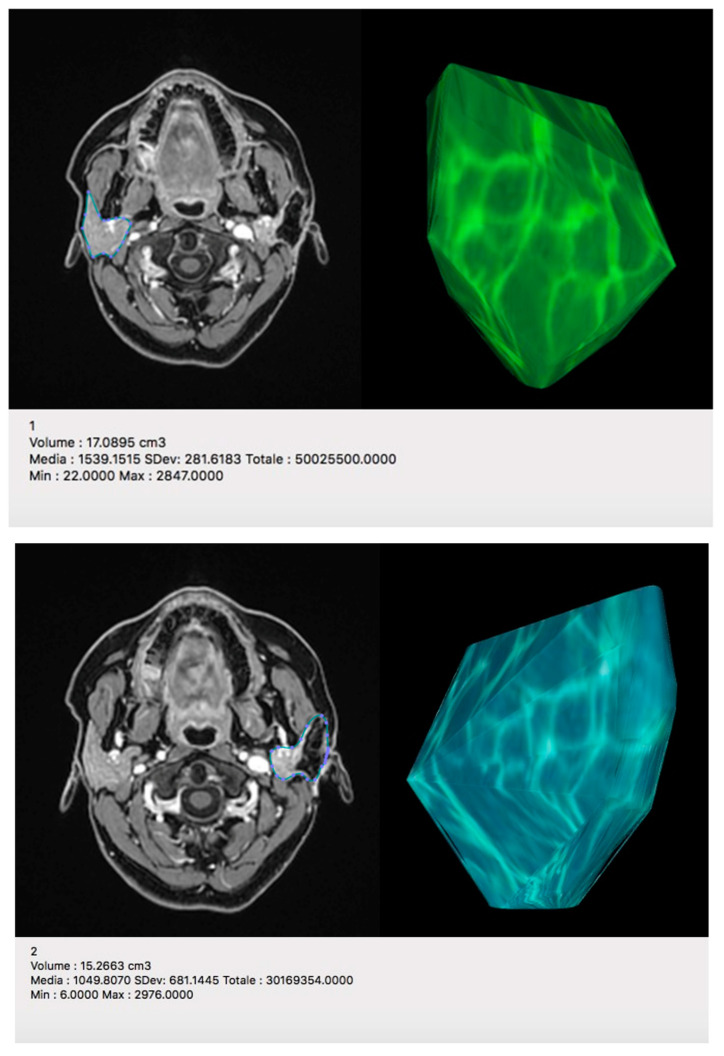
Postoperative volumetric comparison with 3D reconstruction between the contralateral (**1**) and the reconstructed parotid gland (**2**) shows minimal difference in volume (17.1 cm^3^ vs. 15.2 cm^3^).

The volumetric comparison conducted in postoperative studies showed no significant difference in volume between the two parotid glands (Figure 5).

## 5. Conclusions

Based on our satisfactory feedback received from patients about the overall postoperative cosmetic result, and with validation from our radiology team regarding the safety of this surgical procedure even in patients with malignant lesions, we can define fat grafting as a surgical procedure with high rates of success in terms of aesthetic results, the duration of the entire procedure, and its safety profile during the oncological follow up; with our experience, we confirm the already well-known fame of the autologous dermis-fat graft as a milestone in craniofacial reconstructive surgery.

## Figures and Tables

**Table 1:** Patients’ characteristics, including gender, age, parotid first-step surgery (1: partial parotidectomy; 2: superficial parotidectomy; 3: total parotidectomy), and final histologic diagnosis.

Patient	Gender	Age	Extent of Surgery (P.P. ^1^, S.P. ^2^, T.P. ^3^)	Final Histologic Diagnosis
1	F	49	P.P.	Pleomorphic adenoma
2	F	28	P.P.	Pleomorphic adenoma
3	F	14	P.P.	Pleomorphic adenoma
4	F	73	P.P.	Warthin tumour
5	M	54	T.P.	Mucoepidermoid carcinoma
6	M	78	T.P.	Salivary duct carcinoma
7	M	69	T.P.	Metastatic squamous cell carcinoma
8	F	21	T.P.	Acinic cell carcinoma
9	M	51	P.P.	Warthin tumour
10	M	21	P.P.	Pleomorphic adenoma
11	F	64	P.P.	Warthin tumour
12	F	48	P.P.	Warthin tumour
13	F	56	S.P.	Carcinoma ex pleomorphic adenoma
14	F	33	S.P.	Myoepithelioma
15	M	51	T.P.	Salivary duct carcinoma
16	F	37	S.P.	Pleomorphic adenoma (recurrency)
17	M	67	T.P.	Pleomorphic adenoma (recurrency)
18	F	55	T.P.	Pleomorphic adenoma
19	F	59	S.P.	Mucoepidermoid carcinoma (low grade)
20	M	65	P.P.	Solitary Extrapleural Fibrous Tumour
21	F	53	S.P.	Myoepithelioma
22	F	75	S.P.	Myoepithelioma
23	M	63	S.P.	Basal cells adenoma
24	F	37	P.P.	Warthin tumour
25	F	76	S.P.	Myoepithelioma
26	M	45	S.P.	Pleomorphic adenoma
27	F	62	P.P.	Pleomorphic adenoma
28	F	52	P.P.	Warthin tumour
29	F	44	S.P.	Pleomorphic adenoma
30	F	42	S.P.	Acinic cell carcinoma

## Data Availability

Data are available upon reasonable request from the corresponding author (R.V.).
